# Treatment of primary *g*lioblastoma multiforme with c*e*tuximab, *r*adiotherapy and *t*emozolomide (GERT) – phase I/II trial: study protocol

**DOI:** 10.1186/1471-2407-6-133

**Published:** 2006-05-18

**Authors:** Stephanie E Combs, Steffen Heeger, Renate Haselmann, Lutz Edler, Jürgen Debus, Daniela Schulz-Ertner

**Affiliations:** 1 Department of Radiation Oncology, University of Heidelberg, Im Neuenheimer Feld 400, 69120 Heidelberg, Germany; 2 Department of Biostatistics, German Cancer Research Center, Im Neuenheimer Feld 280, 69120 Heidelberg, Germany; 3 Merck KGaA, Frankfurter Str. 250, 64293 Darmstadt, Germany

## Abstract

**Background:**

The implementation of combined radiochemotherapy (RCHT) with temozolomide (TMZ) has lead to a significant increase in overall survival times in patients with Glioblastoma multiforme (GBM), however, outcome still remains unsatisfactory.

The majority of GBMs show an overexpression and/or amplification of the epidermal growth factor receptor (EGFR). Therefore, addition of EGFR-inhibition with cetuximab to the current standard treatment approach with radiotherapy and TMZ seems promising.

**Methods/design:**

GERT is a one-armed single-center phase I/II trial. In a first step, dose-escalation of TMZ from 50 mg/m^2 ^to 75 mg/m^2 ^together with radiotherapy and cetuximab will be performed. Should safety be proven, the phase II trial will be initiated with the standard dose of 75 mg/m^2 ^of TMZ. Cetuximab will be applied in the standard application dose of 400 mg/m^2 ^in week 1, thereafter at a dose of 250 mg/m^2 ^weekly. A total of 46 patients will be included into this phase I/II trial.

Primary endpoints are feasibility and toxicity, secondary endpoints are overall and progression-free survival. An interim analysis will be performed after inclusion of 15 patients into the main study. Patients' enrolment will be performed over a period of 2 years. The observation time will end 2 years after inclusion of the last patient.

**Discussion:**

The goal of this study is to evaluate the safety and efficacy of combined RCHT-immunotherapy with TMZ and cetuximab as first-line treatment for patients with primary GBM.

## Background

Glioblastoma multiforme (GBM) is the most frequent primary malignant brain tumor in adults. Until recently, the standard treatment approach in patients with GBM was neurosurgical resection, as radical as possible, followed by postoperative radiotherapy (RT). However, in spite of technical advances in surgery and radiotherapy, overall survival still remained unsatisfactory with median overall survival times of 9–12 months [1,2].

Over the last decade, a number of clinical investigations on combined radio-chemotherapy (RCHT) after neurosurgical resection have been conducted. A large randomized trial performed by the Neuro-Oncology Working Group of the German Cancer Society evaluated combined RCHT with nimustine plus teniposide versus nimustine plus cytarabine and could obtain a median overall survival time of 16.5 months in patients with GBM [3]. Temozolomide (TMZ), an oral alkylating agent, had demonstrated antitumor activity as a single-agent treatment in recurrent GBM [4-6].

In a pilot phase, the feasibility of concomitant administration of TMZ and fractionated RT followed by 6 cycles of adjuvant TMZ was demonstrated and one could suggest that this combined treatment modality would offer significant benefit for patients with GBM [7]. At the Department of Radiation Oncology at the University of Heidelberg a trial evaluating combined RCHT with TMZ in a dosage of 50 mg/m^2 ^5 days per week was conducted, without adjuvant application of TMZ; we observed a median overall survival time of 19 months, and treatment-related toxicity was low [8].

A large randomized trial conducted by the EORTC evaluated the outcome after combined RCHT with TMZ followed by adjuvant TMZ application as opposed to RT alone; in patients treated with RCHT, overall survival was significantly increased to 14.6 months as compared to RT alone with 12.1 months [9].

Treatment-related toxicity was relatively high in the combined treatment arm with 14% of patients presenting with WHO Grade 3 or 4 hematologic toxicities as compared to 7% in the RT-group. Additionally, RT was interrupted or delayed in 32% of the RCHT-patients, and only 47% of all RCHT patients completed the planned 6 cycles of adjuvant TMZ-application. However, the significant increase in overall survival can be considered a major progress and thus the current standard for patients with GBM is considered RT together with the concomitant and adjuvant application of TMZ.

In spite of these advances in outcome, overall survival is still dissatisfactory. Therefore, novel approaches must be implemented into clinical evaluation. Recently, a number of molecular targeting agents have been developed and evaluated in early clinical trials. The main ulterior motive for these therapies is that by intervening into molecular mechanisms the treatment resistance of cancer cells may be overcome, and an amplification of the RCHT-response might be achieved.

To date, several targets have been identified and include vascular-endothelial growth factor (VEGF), platelet-derived growth factor (PDGF), agents targeting components of the Ras- and Akt-mediated pathways, as well as the human epidermal growth factor receptor (HER). All of these are known to play a key role in tumorigenesis and disease progression [10].

The HER-family consists of four distinct receptors: HER1/EGFR (epidermal growth factor receptor), HER2, HER3 and HER4 [11,12]. The EGFR gene is a proto-oncogene that is often amplified in a variety of human tumors [13,14]. The EGFR-gene is located on chromosome 7 and encodes for a 170 kD transmembrane glycoprotein with intrinsic tyrosine-kinase activity [15,16]. The receptor molecules consist of an extracellular ligand binding domain and an intracellular tyrosine kinase that is activated via conformational change in the intracellular protein domain due to extracellular ligand binding and receptor dimerization; the activation of the tyrosine kinase results in phosphorylation of intracellular substrate proteins, kicking off an intracellular reaction cascade regulating cell function and division, apoptosis, adhesion, motility and neoangiogenesis [17]. There are also several mutant HER1/EGFR, the most common one being EGFRvIII, which is constitutively activated and leads to downstream intracellular cascade activation without ligand binding [18-20].

In GBM, the EGFR gene is one of the most frequently amplified proto-oncogenes; expression of the EGFR-protein has been observed in 24–95% of cases [13,14,21] (Table 1).

The real prognostic significance remains unclear to date. However, in a number of other cancers including breast, bladder, esophagus, cervix, ovary, lung and head and neck the overexpression of EGFR has been reported to be a poor prognostic factor [22-24].

A number of small molecules acting as tyrosine-kinase inhibitors (TKIs) have been evaluated in the treatment of GBM. A subgroup of patients demonstrated a response to these agents, however, no correlation with overall survival could be demonstrated [25,26]. A recent publication by Mellinghoff et al. could identify patients expressing EGFRvIII as well as the tumor suppression protein PTEN showing a greater response to the TKIs [25].

Cetuximab is a monoclonal antibody binding specifically to EGFR and thus inhibiting downstream signal transduction pathways [27]. It has been shown that cetuximab enhances radiosensitivity, promotes radiation-induced apoptosis, decreases cell proliferation, inhibits radiation-induced damage repair and inhibits tumor angiogenesis [23].

A number of clinical studies with the EGFR-antibody cetuximab have shown that its application is efficacious and tolerable, with the most common toxicity being acneiform rash. In general, allergic reactions including anaphylaxis could be a potential side effect. A number of phase I and II clinical studies have been conduced with cetuximab as a single agent and have lead to response rates up to 12% and additional stable disease rates up to 32% of all patients with advanced non small cell lung cancer (NSCLC), ovarian cancer, head and neck cancer and colorectal cancer [23]. A major trial evaluating a combination of radiotherapy plus cetuximab versus radiotherapy alone in patients with locoregionally advanced squamous cell carcinoma of the head-and-neck could demonstrate a significant increase in local tumor control rates as well as in overall survival. In a number of the studies conducted, EGFR-overexpression correlated significantly with patients' outcome [22,23,28,29].

Therefore, two main rationales have lead to the initiation of the present study protocol:

1. Overexpression of EGFR has been shown to influence outcome of EGFR-inhibition in patients with extracranial tumors.

2. The majority of GBM show overexpression of EGFR as wildtype or as mutant.

Therefore, the present study protocol evaluates EGFR-inhibition with cetuximab together with the standard radiochemotherapeutic treatment consisting of RCHT with TMZ for patients with primary GBM.

## Methods/design

### Study design

The study is designed as a one-armed study evaluating the feasibility and toxicity of combined radiochemo-immunotherapy with TMZ and cetuximab. The trial can be divided into two phases: During the Phase I part, dose escalation of TMZ from 50 mg/m^2 ^to 75 mg/m^2 ^together with RT and cetuximab will be performed. Thereafter, the Phase II part of the trial will be initiated with a daily total dose of 75 mg/m^2 ^with concomitant RT and cetuximab.

### Study objectives

Primary endpoint of the study is feasibility and toxicity of combined trimodal therapy with TMZ, cetuximab and radiotherapy. According to the current standard treatment for patients with GBM, about 10% off all patients develop WHO Grade III and IV toxicity [9]. We defined a toxicity rate of 30% for WHO Grade III and IV side effects as overall acceptable for this study.

Secondary endpoints include overall survival, progression-free survival and quality of life.

### Trial organization

The study protocol of GERT was designed by the study initiators at the Department of Radiation Oncology at the University of Heidelberg. It is an investigator-initiated trial (IIT); trial medication is provided by Merck KgaA in Darmstadt, Germany. The trial is conducted at the Department of Radiation Oncology at the University of Heidelberg.

### Coordination

The overall coordination is performed by the Department of Radiation Oncology at the University of Heidelberg, and this department is responsible for overall trial management, trial registration, database management and quality assurance. The study monitoring according to the guidelines of Good Clinical Practice (GCP) is performed by the Koordinierungszentrum für Klinische Studien (KKS), University of Heidelberg. Biometrical data analysis is conducted together with the Department of Biostatistics of the German Cancer Research Center (dkfz).

### Investigators

The study investigators are experienced radiation oncologists specialized in the treatment of patients with GBM. Patients will be recruited and treated by the physicians of the Department of Radiation Oncology of the University of Heidelberg.

### Patient selection

Inclusion criteria into the study protocol include:

- patients ≥18 and < 70 years of age

- Karnofsky Performance Score ≥60

- histologically confirmed supratentorial GBM

- interval between primary diagnosis and registration for the study < 4 weeks

- patients will be included according to the incidental gender distribution for patients with GBM of ♀/♂ 2:3

- adequate blood values (not older than 14 days prior to initiation of RCHT)

- neutrophil count (ANC) ≥1500/mm3 or white blood cells (WBC) ≥2000/mm3

- platelets ≥100.000/mm3

- hemoglobin ≥10 g/dL

- BUN <1.5 times the upper range

- Total and direct bilirubin <1.5 times the upper laboratory limit

- Adequate liver enzymes <3 times the upper laboratory limit

- Life expectancy >12 weeks

- Written informed consent

Additionally, the combined treatment with radiotherapy and TMZ would be the standard treatment recommended to be performed in the patients included into the study.

### Exclusion criteria

• refusal of the patients to take part in the study

• previous radiotherapy of the brain or chemotherapy with DTIC or TMZ

• known allergy against extrinsical proteins

• previous chemotherapy or therapy with an EGFR-inhibitor

• Previous antibody therapy

• Patients who have not yet recovered from acute toxicities of prior therapies

• Acute infections requiring systemic application of antibiotics

• Frequent vomiting or a medical condition preventing the oral application of TMZ

• Clinically active kidney-liver or cardiac disease

• Known carcinoma < 5 years ago (excluding Carcinoma in situ of the cervix, basal cell carcinoma, squamous cell carcinoma of the skin)

• HIV

• Pregnant or lactating women

• Participation in another clinical study

### Statistical calculations for trial sample size

The trial is evaluating in its Phase 1 part the tolerability of the TMZ dose of 75 mg/m^2 ^together with cetuximab in a two step escalation with a maximum of 12 patients, while evaluation in its Phase II part the following two hypotheses:

1. non-tolerable toxicity H0: existence of no tolerable toxicity, i.e. TR >30%, NTR ≤70%

2. tolerable toxicity H1: existence of tolerable toxicity, i.e. TR < 10%, NTR ≥90%

with RT denoting the rate of toxicity and NTR the rate of non-toxicity. Therefore, the trial consists of a dose finding part (Phase I) and a feasibility part (Phase II).

These two hypothesis will be examined using a two-step optimal phase II design according to Simon et al. [30] in the defined patient population with a significance level of 5% (alpha = 0.05) and a power of 90% (beta = 0.1) with a one-armed analysis in this single arm trial.

To demonstrate tolerability of the toxicity with a hypothesis-test between NONTOX of 70% versus the anticipated NONTOX of 90% within a One-Step-Phase II Design, 36 evaluable patients are required (NCSS PASS 2000).

We calculated a drop-out rate of 10%, and therefore a projected patient number of 40 is planned.

With inclusion of the 6 patients for the dose-finding part of the protocol, a maximum of 46 patients will be included into the study.

Recruitment will be carried out over 2 years and a follow-up time of 1 year per patient is required to evaluate the primary endpoint.

Overall survival will be determined as the time span between primary diagnosis and death of lost to follow-up (censored observation). Progression-free survival will be determined as the time span between start of the treatment and tumor progression or death, whatever occurred first or an event which characterises a censored observation.

The trial consists of a dose-finding study (part 1; Phase I) followed by a Phase II component.

#### Step 1

3 patients are treated with combined RCHT (RT at dose level 1 (DL1) with a total dose of 60 Gy in 2 Gy single fractions + TMZ 50 mg/m^2^/day, 6 weeks)

Cetuximab (ERBITUX^®^) is applied in a dose of 400 mg/m^2 ^in week 1, thereafter weekly at 250 mg/m^2 ^week during the course of RCHT.

Adjuvant treatment: 6 cycles of TMZ 150 mg/m^2^/day for 5 days, every 28 days (q28d)

If 2 out of 3 patients develop DLT, the study will be stopped. If 1 out of 3 patients develops DLT, three more patients will be included at a dose of 50 mg/m^2 ^TMZ. Dose escalation is halted if two ore more patients develop DLT.

#### Step 2

3 Patients are treated with combined RCHT (RT with GD 60 Gy, ED 2.0 Gy + TMZ 75 mg/m^2^/day, 6 weeks)

Cetuximab (ERBITUX^®^) 400 mg/m^2 ^in week 1, thereafter at a dose of 250 mg/m^2 ^weekly during RCHT.

Adjuvant treatment: 6 cycles of TMZ 150 mg/m^2^/day for 5 days, every 28 days (q28d).

If DLT occurs in one patient additional three patients will be treated at DL 2. If 2 or more patients develop DLT, DL 1 will be the recommended dose for the phase II part of the study the study.

### Adverse events

The investigators will report all Adverse Events (AE) immediately to the sponsor or his legal representative except for those that the protocol or investigator's brochure identifies as not requiring immediate reporting.

An "adverse event" is any untoward medical occurrence in a patient or clinical trial subject administered a medicinal product and which does not necessarily have a causal relationship with this treatment.

Serious adverse events (SAE) are any untoward medical occurrence of effect that at any dose results in death, is life-threatening, requires hospitalization or prolongation of existing inpatients' hospitalization, results in persisting or significant disability or incapability, or is a congenital abnormality or birth defect.

For all serious adverse events, the documents and patient data must be verified by the responsible study personnel.

Patients' toxicities are to be classified and documented according to the NCI Common Toxicity Criteria (CTC). Toxicity is documented weekly during RCHT and prior to every application of Cetuximab as well as at every follow-up visit.

### Medication

All TMZ applications are provided by the pharmacy of the University of Heidelberg. Cetuximab is provided by Merck KGaA, Darmstadt, and is stored until clinical application by the pharmacy of the University of Heidelberg. All study medication is prepared especially for each patient registered into the study and is delivered to the Department of Radiation Oncology at short notice before administration.

### Monitoring

Monitoring is performed by the Klinische Koordinierunszentrum für Studien (KKS) of the University Hospital in Heidelberg according the guidelines of Good Clinical Practice (GCP) and the German law on drug safety (Arzneimittelgesetz, AMG).

### Ethics, informed consent and safety

For the present study the Eudract-Number 2005-003911-63 has been obtained. The final study protocol has been approved by the ethics committee of the University of Heidelberg, Heidelberg, Germany (Protocol Number AFmo-323/2005 [31]) as well as by the Paul Ehrlich Institute (PEI; registration number 119/01). This protocol follows all requirements of the Declaration of Helsinki in the recent German version (Somerset West Version, 1996).

The study protocol is in accordance with the principles of Good Clinical Practice (GCP) guidelines and the Federal Data Protection Act. The trial has been initiated and will be carried out following all local legal and regulatory requirements. The medical secrecy act is also followed.

Of each patient recruited into the study, written informed consent is essential prior to inclusion into the study after extensive information about the intent of the study, the study regimen, potential associated risks and side effects as well as potential alternative therapies. Until the patient has given written informed consent, no action aiming at inclusion into the study protocol, especially any diagnostic measures required, will be taken.

### Quality of life-assessment

Assessment of quality of life is one of the secondary objectives of the trial. The EORTC QLQ30 is a general measure of quality of life in cancer patients. The questionnaire consists of 9 multi-item scales: five functional scales (physical, role, cognitive, emotional and social), three symptom scales (fatigue, pain and nausea/vomiting) as well as global health and quality of life scale [32]. Specific symptoms including dyspnoea, insomnia, anorexia, constipation, diarrhoea and financial impact are measure as six single items. This questionnaire has been used widely in studies with cancer patients and is able to distinguish between individuals with metastatic and non-metastatic disease, as well as between patients at different stages of their illness. Previous studies have proven this tool to have good internal consistency (alpha > 0.70) and good test-re-test-reliability (0.80 to 0.90) [33].

This test will be performed at the initiation of RCHT, during the course of the treatment and at every follow-up visit.

### Follow-up

Patients are seen for follow-up 6 weeks after completion of RCHT with cetuximab, thereafter in three-months intervals or as needed clinically. During adjuvant treatment, blood values will be evaluated weekly.

All follow-up visits include complete neurological assessment as well as contrast-enhanced MRI- and/or CT-scans. Quality of life assessment will be performed at each follow-up visit.

Additional examinations including FDG- or FLT-PET will be scheduled as required.

### Treatment at tumor progression

In order to distinguish between radiation-induced side effects and tumor progression, FDG-PET and FLT-PET examinations will be scheduled as required.

In case of tumor recurrence during combined RCHT with cetuximab or during adjuvant treatment with TMZ, this therapy will be stopped.

In every case of recurrence, the possibility of neurosurgical intervention will be evaluated. Additionally, it will be evaluated whether re-irradiation can be applied safely; moreover, treatment with standard chemotherapeutic substances will be evaluated in every patient.

### Interim analysis

An interim analysis is planned after inclusion of 15 patients into the main study. The trial can be stopped should it be obvious that the alternative hypothesis of a tolerable toxicity rate

H1:R≤10% i.e. NONTOX-Rate ≥90%

cannot be achieved, i.e. should the number of WHO grade III and IV toxicities be larger than 7 (meaning 8 or more Grade III and IV toxicities). In this case the study will be closed due to "futility".

Recruitment for the study will be conducted during a period of 2 years. Final evaluation of the primary and secondary endpoints of the study will be performed 1 year after inclusion of the last patient.

The formal end of the study is defined as the end of the observation period of the last patient included into the study.

## Discussion

The trial is carried out as a single center trial at the University of Heidelberg, Department of Radiation Oncology in Germany.

The department has a strong focus and neuro-oncology [8,34-46]. The department offers all techniques of conventional RT as well as all modern forms of high-precision RT including fractionated stereotactic radiotherapy (FSRT) and intensity modulated radiotherapy (IMRT) [47-53]. Extensive experience in combined multimodality treatment approaches is provided as well as three wards for patients requiring hospitalization. All other departments of the University Hospital of Heidelberg including neurosurgery and nuclear medicine are in close vicinity offering all medial care potentially needed. Therefore, all essential requirements to conduct the present study are met at our department.

With the present trial, we aim at improving outcome in patients with GBM by adding the promising molecular target of EGFR-inhibition with cetuximab to the current treatment standard for patients with GBM consisting of radiotherapy combined with TMZ.

## Abbreviations

CRO Clinical Research Organization

EGFR Epidermal Growth Factor Receptor

GBM Glioblastoma multiforme

GCP Good Clinical Practice

RCHT Radio-Chemotherapy

TMZ Temozolomide

## Competing interests

The author(s) declare that they have no competing interests.

## Authors' contributions

SEC, JD and DSE planned, organized and conduct the study. SH supported project management and protocol development. Medical care is provided by SEC, JD and DSE. SEC and DSE recruit the patients for the study. LE performed statistical calculations for the planning of the study and will be involved in interim and final statistical analyses. RH was involved in establishment of the study protocol and coordinates study performance.

All authors have read and have approved the final manuscript.

## Pre-publication history

The pre-publication history for this paper can be accessed here:



## Figures and Tables

**Table 1:** Percentage of reported EGFR-amplification/overexpression in patients with glioblastoma multiforme: Literature overview. Abbreviations: Southern Blot (SB), Immunohistochemistry (IHC), Western Blot (WB), Polymerase Chain Reaction (PCR), In situ hybridization (ISH), Fluorescence In-Situ Hybridization (FISH).

**Author (Reference)**	**Year of publication**	**Patients (n)**	**Patients with EGFR-amplification (n)**	**Patients with EGFR-overexpression (n)**	**Method of EGFR evaluation**
Quan et al.	2005	107	36 (32%)	-	ISH
Mellinghoff et al.	2005	25	12 (48%)	-	FISH, PCR
Shinojima et al.	2003	87	40 (46%)	-	SB, IHC
Schmidt et al.	2002	91	26 (29%)	-	PCR
Barker et al.	2001	162	-	53 (33%)	IHC
Simmons et al.	2001	110	-	39 (35%)	IHC
Chakravarti et al.	2001	56	-	23 (41%)	WB
Smith et al.	2001	111	46 (41%)	-	FISH, PCR
Yoon et al.	2001	36	13 (36%)	12 (33%)	PCR, IHC
Korshunov et al.	1999	168	-	102 (61%)	IHC
Olson et al.	1998	70	19 (27%)	-	SB
Bouvier-Labit et al.	1998	63	-	22 (35%)	IHC
Newcomb et al.	1998	80	-	28 (35%)	IHC
Korkopopoulou et al.	1997	37	-	17 (64%)	IHC
Rainov et al.	1997	71	-	54 (76%)	IHC
Liu et al.	1997	63	31 (49%)	-	SB
Waha et al.	1996	54/62	18 (33%)	32 (52%)	PCR, IHC
Zhu et al.	1996	55	-	39 (71%)	IHC
Diedrich et al.	1995	32/22	13 (41%)	19 (86%)	ISH
Kordek et al.	1995	30	-	10 (33%)	IHC
Schober et al.	1995	21	13 (62%)	-	PCR, IHC
Schlegel et al.	1994	42	19 (45%)	27 (64%)	SB, PCR, WB
Hiesinger et al.	1993	17	-	4 (24%)	IHC
Hurtt et al.	1992	19	6 (32%)	-	Dot blot
Jaros et al.	1992	20	-	19 (95%)	IHC
Torp et al.	1992	20	8 (40%)	-	SB
Bigner et al.	1988	48	20 (42%)	NR	SB, slot blot
Wong et al.	1987	47	20 (43%)	-	SB, slot blot
